# 5-Bromo-4-iodo-2-methyl­aniline

**DOI:** 10.1107/S160053681200921X

**Published:** 2012-03-10

**Authors:** Yan-Ju Liu, Da-Shun Dai

**Affiliations:** a Pharmacy College, Henan University of Traditional Chinese Medicine, Zhengzhou 450008, People’s Republic of China; b Henan Hospital of Traditional Chinese Medicine, Zhengzhou 450008, People’s Republic of China

## Abstract

The asymmetric unit of the title compound, C_7_H_7_BrIN, contains two independent mol­ecules, which are linked by weak N—H⋯N hydro­den-bonding inter­actions between the amino groups.

## Related literature

For the synthetic procedure, see: Lee *et al.* (2005 ▶). For bond-length data, see: Allen *et al.* (1987 ▶).
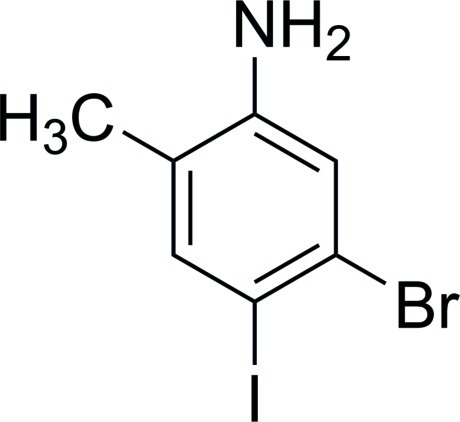


## Experimental

### 

#### Crystal data

C_7_H_7_BrIN
*M* *_r_* = 311.94
Monoclinic, 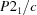

*a* = 26.831 (5) Å
*b* = 5.3920 (11) Å
*c* = 12.217 (2) Å
β = 98.05 (3)°
*V* = 1750.1 (6) Å^3^
*Z* = 8
Mo *K*α radiation
μ = 8.15 mm^−1^
*T* = 293 K
0.20 × 0.10 × 0.10 mm

#### Data collection

Enraf–Nonius CAD-4 diffractometer
Absorption correction: ψ scan (North *et al.*, 1968 ▶) *T*_min_ = 0.292, *T*_max_ = 0.496
3177 measured reflections
3177 independent reflections
2057 reflections with *I* > 2σ(*I*)
3 standard reflections every 200 reflections intensity decay: 1%

#### Refinement

*R*[*F* ^2^ > 2σ(*F* ^2^)] = 0.060
*wR*(*F* ^2^) = 0.163
*S* = 1.01
3177 reflections
183 parameters
H-atom parameters constrained
Δρ_max_ = 0.97 e Å^−3^
Δρ_min_ = −1.03 e Å^−3^

### 

Data collection: *CAD-4 Software* (Enraf–Nonius, 1989) ▶; cell refinement: *CAD-4 Software*; data reduction: *XCAD4* (Harms & Wocadlo, 1995 ▶); program(s) used to solve structure: *SHELXS97* (Sheldrick, 2008 ▶); program(s) used to refine structure: *SHELXL97* (Sheldrick, 2008 ▶); molecular graphics: *SHELXTL* (Sheldrick, 2008 ▶); software used to prepare material for publication: *SHELXTL*.

## Supplementary Material

Crystal structure: contains datablock(s) I, LYJ. DOI: 10.1107/S160053681200921X/aa2048sup1.cif

Structure factors: contains datablock(s) I. DOI: 10.1107/S160053681200921X/aa2048Isup2.hkl

Supplementary material file. DOI: 10.1107/S160053681200921X/aa2048Isup3.cml

Additional supplementary materials:  crystallographic information; 3D view; checkCIF report

## Figures and Tables

**Table 1:** Hydrogen-bond geometry (Å, °)

*D*—H⋯*A*	*D*—H	H⋯*A*	*D*⋯*A*	*D*—H⋯*A*
N2—H2*A*⋯N1	0.86	2.67	3.300 (15)	131

**Fig. 1.**
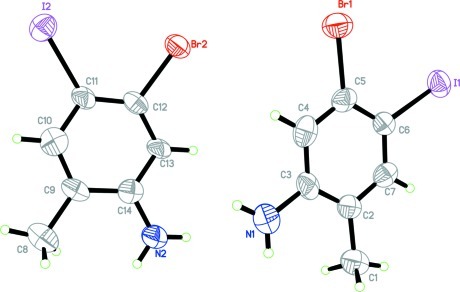
The molecular structure of (I), with the atom-numbering scheme. Displacement ellipsoids are drawn at the 50% probability level.

**Fig. 2.**
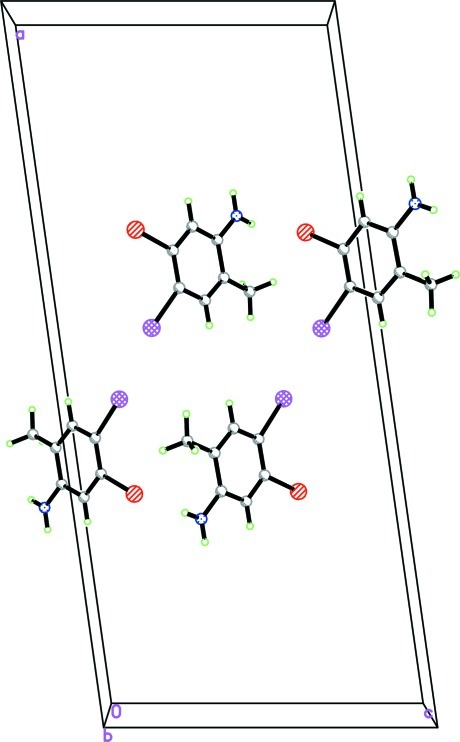
A packing diagram of (I).

## supplementary crystallographic information

### Comment

The tittle compound, 5-bromo-4-iodo-2-methylaniline is an important intermediate, which can be utilized to synthesize highly fluorescent solid-state asymmetric spirosilabifluorene derivatives (Lee *et al.*, 2005). And we report here the crystal structure of the title compound (I), see Fig. 1.

The asymmetric unit contains two title molecules of 5-bromo-4-iodo-2-methylaniline. Weak hydroden bonding interactions link them together with N···N distance 3.300 (14) Å. The bromo, iodo and amino substituents lie in the mean plane of the phenyl rings, with mean deviation of 0.0040 (1) Å from the plane (C2—C7), and 0.0120 (1) Å from the plane (C9—C14). The bond lengths and angles are within normal ranges (Allen *et al.*, 1987).

### Experimental

The title compound, (I) was prepared by a method reported in literature (Lee *et al.*, 2005). The crystals were obtained by dissolving (I) (0.5 g) in methanol (50 ml) and evaporating the solvent slowly at room temperature for about 10 d.

### Refinement

H atoms were positioned geometrically and refined as riding, with N—H = 0.86 Å and C—H = 0.93 Å, with *U*_iso_(H) = 1.2*U*_eq_(C, N).

### Crystal data

**Table:**

C_7_H_7_BrIN	*F*(000) = 1152
*M**_r_* = 311.94	*D*_x_ = 2.368 Mg m^−^^3^
Monoclinic, *P*2_1_/*c*	Mo *K*α radiation, λ = 0.71073 Å
Hall symbol: -P 2ybc	Cell parameters from 25 reflections
*a* = 26.831 (5) Å	θ = 9–13°
*b* = 5.3920 (11) Å	µ = 8.15 mm^−^^1^
*c* = 12.217 (2) Å	*T* = 293 K
β = 98.05 (3)°	Block, colourless
*V* = 1750.1 (6) Å^3^	0.20 × 0.10 × 0.10 mm
*Z* = 8	

### Data collection

**Table:**

Enraf–Nonius CAD-4 diffractometer	2057 reflections with *I* > 2σ(*I*)
Radiation source: fine-focus sealed tube	*R*_int_ = 0.000
Graphite monochromator	θ_max_ = 25.3°, θ_min_ = 1.5°
ω/2θ scans	*h* = −32→31
Absorption correction: ψ scan (North *et al.*, 1968)	*k* = 0→6
*T*_min_ = 0.292, *T*_max_ = 0.496	*l* = 0→14
3177 measured reflections	3 standard reflections every 200 reflections
3177 independent reflections	intensity decay: 1%

### Refinement

**Table:**

Refinement on *F*^2^	Primary atom site location: structure-invariant direct methods
Least-squares matrix: full	Secondary atom site location: difference Fourier map
*R*[*F*^2^ > 2σ(*F*^2^)] = 0.060	Hydrogen site location: inferred from neighbouring sites
*wR*(*F*^2^) = 0.163	H-atom parameters constrained
*S* = 1.01	*w* = 1/[σ^2^(*F*_o_^2^) + (0.0946*P*)^2^] where *P* = (*F*_o_^2^ + 2*F*_c_^2^)/3
3177 reflections	(Δ/σ)_max_ < 0.001
183 parameters	Δρ_max_ = 0.97 e Å^−^^3^
0 restraints	Δρ_min_ = −1.03 e Å^−^^3^

### Special details

**Table:**

Geometry. All e.s.d.'s (except the e.s.d. in the dihedral angle between two l.s. planes) are estimated using the full covariance matrix. The cell e.s.d.'s are taken into account individually in the estimation of e.s.d.'s in distances, angles and torsion angles; correlations between e.s.d.'s in cell parameters are only used when they are defined by crystal symmetry. An approximate (isotropic) treatment of cell e.s.d.'s is used for estimating e.s.d.'s involving l.s. planes.
Refinement. Refinement of *F*^2^ against ALL reflections. The weighted *R*-factor *wR* and goodness of fit *S* are based on *F*^2^, conventional *R*-factors *R* are based on *F*, with *F* set to zero for negative *F*^2^. The threshold expression of *F*^2^ > σ(*F*^2^) is used only for calculating *R*-factors(gt) *etc*. and is not relevant to the choice of reflections for refinement. *R*-factors based on *F*^2^ are statistically about twice as large as those based on *F*, and *R*- factors based on ALL data will be even larger.

### Fractional atomic coordinates and isotropic or equivalent isotropic displacement parameters (Å^2^)

**Table:**

	*x*	*y*	*z*	*U*_iso_*/*U*_eq_	
I1	0.05030 (3)	−0.37033 (14)	0.72451 (7)	0.0542 (3)	
Br1	0.18159 (5)	−0.3594 (2)	0.82076 (10)	0.0583 (4)	
N1	0.2022 (4)	0.3506 (17)	0.5418 (9)	0.058 (3)	
H1A	0.2337	0.3468	0.5679	0.069*	
H1B	0.1914	0.4538	0.4902	0.069*	
C1	0.0968 (5)	0.373 (2)	0.4509 (9)	0.053 (3)	
H1C	0.1125	0.3476	0.3859	0.080*	
H1D	0.1026	0.5404	0.4765	0.080*	
H1E	0.0612	0.3447	0.4336	0.080*	
C2	0.1187 (4)	0.1958 (19)	0.5397 (9)	0.041 (3)	
C3	0.1691 (4)	0.191 (2)	0.5825 (9)	0.043 (3)	
C4	0.1864 (4)	0.025 (2)	0.6653 (10)	0.049 (3)	
H4A	0.2204	0.0251	0.6940	0.059*	
C5	0.1542 (4)	−0.1415 (19)	0.7070 (9)	0.042 (3)	
C6	0.1033 (4)	−0.1352 (17)	0.6651 (8)	0.036 (2)	
C7	0.0864 (4)	0.030 (2)	0.5818 (9)	0.044 (3)	
H7A	0.0524	0.0309	0.5530	0.052*	
I2	0.45125 (3)	−0.34995 (15)	0.68253 (7)	0.0534 (3)	
Br2	0.31999 (5)	−0.3860 (2)	0.68824 (9)	0.0502 (3)	
N2	0.2915 (4)	0.2963 (19)	0.3831 (9)	0.060 (3)	
H2A	0.2599	0.2726	0.3840	0.072*	
H2B	0.3014	0.4056	0.3395	0.072*	
C8	0.3973 (6)	0.385 (2)	0.3750 (11)	0.067 (4)	
H8A	0.3858	0.5453	0.3943	0.100*	
H8B	0.3841	0.3482	0.2996	0.100*	
H8C	0.4334	0.3837	0.3836	0.100*	
C9	0.3795 (4)	0.1937 (19)	0.4488 (9)	0.044 (3)	
C10	0.4119 (5)	0.039 (2)	0.5159 (9)	0.048 (3)	
H10A	0.4463	0.0539	0.5135	0.057*	
C11	0.3960 (4)	−0.137 (2)	0.5865 (8)	0.041 (3)	
C12	0.3454 (4)	−0.1580 (18)	0.5898 (8)	0.038 (2)	
C13	0.3108 (4)	−0.019 (2)	0.5224 (8)	0.040 (3)	
H13A	0.2765	−0.0421	0.5237	0.048*	
C14	0.3270 (4)	0.157 (2)	0.4527 (9)	0.043 (3)	

### Atomic displacement parameters (Å^2^)

**Table:**

	*U*^11^	*U*^22^	*U*^33^	*U*^12^	*U*^13^	*U*^23^
I1	0.0587 (5)	0.0456 (5)	0.0631 (6)	−0.0017 (4)	0.0249 (4)	0.0019 (4)
Br1	0.0684 (8)	0.0578 (8)	0.0482 (8)	0.0159 (6)	0.0067 (6)	0.0058 (6)
N1	0.050 (6)	0.065 (7)	0.061 (7)	−0.002 (5)	0.015 (5)	0.007 (6)
C1	0.073 (8)	0.045 (7)	0.043 (7)	−0.002 (6)	0.013 (6)	−0.003 (6)
C2	0.053 (7)	0.037 (6)	0.033 (6)	0.004 (5)	0.012 (5)	−0.006 (5)
C3	0.050 (7)	0.050 (7)	0.034 (6)	0.002 (6)	0.020 (5)	−0.006 (5)
C4	0.046 (7)	0.050 (7)	0.053 (7)	0.008 (6)	0.013 (6)	−0.017 (6)
C5	0.050 (6)	0.030 (6)	0.046 (6)	0.011 (5)	0.008 (5)	−0.004 (5)
C6	0.046 (6)	0.025 (5)	0.038 (6)	−0.001 (5)	0.012 (5)	−0.008 (5)
C7	0.050 (7)	0.041 (6)	0.039 (6)	0.008 (5)	0.003 (5)	−0.010 (5)
I2	0.0509 (5)	0.0515 (5)	0.0565 (5)	0.0043 (4)	0.0028 (4)	−0.0006 (4)
Br2	0.0534 (7)	0.0530 (8)	0.0453 (7)	−0.0088 (6)	0.0104 (5)	0.0087 (6)
N2	0.052 (6)	0.063 (7)	0.061 (7)	−0.006 (5)	0.002 (5)	0.024 (6)
C8	0.082 (10)	0.056 (9)	0.064 (9)	−0.014 (7)	0.019 (7)	0.008 (7)
C9	0.064 (8)	0.032 (6)	0.039 (6)	−0.001 (5)	0.020 (6)	0.002 (5)
C10	0.060 (7)	0.052 (7)	0.033 (6)	−0.002 (6)	0.009 (5)	−0.016 (6)
C11	0.046 (6)	0.051 (7)	0.028 (5)	−0.002 (5)	0.016 (5)	0.003 (5)
C12	0.053 (6)	0.036 (6)	0.028 (5)	−0.006 (5)	0.015 (5)	−0.005 (5)
C13	0.050 (7)	0.046 (7)	0.025 (5)	0.000 (5)	0.012 (5)	0.007 (5)
C14	0.050 (6)	0.040 (6)	0.042 (6)	0.007 (5)	0.012 (5)	−0.005 (5)

### Geometric parameters (Å, º)

**Table:**

I1—C6	2.108 (10)	I2—C11	2.098 (11)
Br1—C5	1.889 (11)	Br2—C12	1.911 (10)
N1—C3	1.378 (14)	N2—C14	1.405 (14)
N1—H1A	0.8600	N2—H2A	0.8600
N1—H1B	0.8600	N2—H2B	0.8600
C1—C2	1.503 (15)	C8—C9	1.490 (14)
C1—H1C	0.9600	C8—H8A	0.9600
C1—H1D	0.9600	C8—H8B	0.9600
C1—H1E	0.9600	C8—H8C	0.9600
C2—C3	1.381 (15)	C9—C10	1.388 (15)
C2—C7	1.392 (15)	C9—C14	1.430 (15)
C3—C4	1.381 (16)	C10—C11	1.391 (15)
C4—C5	1.393 (16)	C10—H10A	0.9300
C4—H4A	0.9300	C11—C12	1.369 (15)
C5—C6	1.390 (14)	C12—C13	1.375 (15)
C6—C7	1.380 (14)	C13—C14	1.383 (15)
C7—H7A	0.9300	C13—H13A	0.9300
			
C3—N1—H1A	120.0	C14—N2—H2A	120.0
C3—N1—H1B	120.0	C14—N2—H2B	120.0
H1A—N1—H1B	120.0	H2A—N2—H2B	120.0
C2—C1—H1C	109.5	C9—C8—H8A	109.5
C2—C1—H1D	109.5	C9—C8—H8B	109.5
H1C—C1—H1D	109.5	H8A—C8—H8B	109.5
C2—C1—H1E	109.5	C9—C8—H8C	109.5
H1C—C1—H1E	109.5	H8A—C8—H8C	109.5
H1D—C1—H1E	109.5	H8B—C8—H8C	109.5
C3—C2—C7	118.5 (10)	C10—C9—C14	115.8 (10)
C3—C2—C1	123.2 (11)	C10—C9—C8	123.1 (12)
C7—C2—C1	118.3 (10)	C14—C9—C8	121.1 (11)
N1—C3—C4	120.1 (11)	C9—C10—C11	123.7 (11)
N1—C3—C2	120.0 (11)	C9—C10—H10A	118.1
C4—C3—C2	119.9 (11)	C11—C10—H10A	118.1
C3—C4—C5	121.7 (11)	C12—C11—C10	117.8 (10)
C3—C4—H4A	119.2	C12—C11—I2	124.5 (8)
C5—C4—H4A	119.2	C10—C11—I2	117.7 (8)
C6—C5—C4	118.5 (10)	C11—C12—C13	121.9 (10)
C6—C5—Br1	123.2 (8)	C11—C12—Br2	120.8 (8)
C4—C5—Br1	118.3 (9)	C13—C12—Br2	117.3 (8)
C7—C6—C5	119.5 (10)	C12—C13—C14	119.8 (10)
C7—C6—I1	118.3 (8)	C12—C13—H13A	120.1
C5—C6—I1	122.2 (8)	C14—C13—H13A	120.1
C6—C7—C2	122.0 (10)	C13—C14—N2	119.5 (11)
C6—C7—H7A	119.0	C13—C14—C9	120.9 (10)
C2—C7—H7A	119.0	N2—C14—C9	119.6 (10)
			
C7—C2—C3—N1	179.2 (10)	C14—C9—C10—C11	−2.2 (16)
C1—C2—C3—N1	−1.6 (16)	C8—C9—C10—C11	178.9 (11)
C7—C2—C3—C4	−0.2 (16)	C9—C10—C11—C12	−0.1 (16)
C1—C2—C3—C4	179.0 (10)	C9—C10—C11—I2	−179.3 (8)
N1—C3—C4—C5	−178.8 (10)	C10—C11—C12—C13	2.9 (16)
C2—C3—C4—C5	0.6 (16)	I2—C11—C12—C13	−178.0 (8)
C3—C4—C5—C6	−1.4 (16)	C10—C11—C12—Br2	−177.6 (8)
C3—C4—C5—Br1	−179.7 (8)	I2—C11—C12—Br2	1.4 (13)
C4—C5—C6—C7	1.7 (15)	C11—C12—C13—C14	−3.2 (16)
Br1—C5—C6—C7	180.0 (7)	Br2—C12—C13—C14	177.4 (8)
C4—C5—C6—I1	−178.1 (7)	C12—C13—C14—N2	179.2 (10)
Br1—C5—C6—I1	0.1 (12)	C12—C13—C14—C9	0.6 (16)
C5—C6—C7—C2	−1.4 (15)	C10—C9—C14—C13	1.9 (15)
I1—C6—C7—C2	178.5 (8)	C8—C9—C14—C13	−179.1 (11)
C3—C2—C7—C6	0.6 (16)	C10—C9—C14—N2	−176.6 (10)
C1—C2—C7—C6	−178.6 (9)	C8—C9—C14—N2	2.3 (16)

### Hydrogen-bond geometry (Å, º)

**Table:**

*D*—H···*A*	*D*—H	H···*A*	*D*···*A*	*D*—H···*A*
N2—H2*A*···N1	0.86	2.67	3.300 (15)	131
